# A high-efficiency scar-free genome-editing toolkit for *Acinetobacter baumannii*

**DOI:** 10.1093/jac/dkac328

**Published:** 2022-10-11

**Authors:** Rubén de Dios, Kavita Gadar, Ronan R McCarthy

**Affiliations:** Division of Biosciences, Department of Life Sciences, Centre of Inflammation Research and Translational Medicine, College of Health and Life Sciences, Brunel University London, Uxbridge, UB8 3PH, UK; Division of Biosciences, Department of Life Sciences, Centre of Inflammation Research and Translational Medicine, College of Health and Life Sciences, Brunel University London, Uxbridge, UB8 3PH, UK; Division of Biosciences, Department of Life Sciences, Centre of Inflammation Research and Translational Medicine, College of Health and Life Sciences, Brunel University London, Uxbridge, UB8 3PH, UK

## Abstract

**Background:**

The current mutagenesis tools for *Acinetobacter baumannii* leave selection markers or residual sequences behind, or involve tedious counterselection and screening steps. Furthermore, they are usually adapted for model strains, rather than for MDR clinical isolates.

**Objectives:**

To develop a scar-free genome-editing tool suitable for chromosomal and plasmid modifications in MDR *A. baumannii* AB5075.

**Methods:**

We prove the efficiency of our adapted genome-editing system by deleting the multidrug efflux pumps *craA*, *cmlA5* and resistance island 2 (RI2), as well as curing plasmid p1AB5075, and combining these mutations. We then characterized the susceptibility of the mutants compared with the WT to different antibiotics (i.e. chloramphenicol, amikacin and tobramycin) by disc diffusion assays and determined the MIC for each strain.

**Results:**

We successfully adapted the genome-editing protocol to *A. baumannii* AB5075, achieving a double recombination frequency close to 100% and routinely securing the construction of a mutant within 10 working days. Furthermore, we show that both CraA and p1AB5075 are involved in chloramphenicol resistance, and that RI2 and p1AB5075 play a role in resistance to amikacin and tobramycin.

**Conclusions:**

We have developed a versatile and highly efficient genome-editing tool for *A. baumannii.* We have demonstrated it can be used to modify both the chromosome and native plasmids. By challenging the method, we show the role of CraA and p1AB5075 in antibiotic resistance.

## Introduction

*Acinetobacter baumannii* is an aerobic Gram-negative bacterium that is widespread in the environment and inhabits different niches.^1–3^ However, it can also be an opportunistic pathogen that infects immunocompromised patients.^3,4^ Nowadays, it is estimated that up to 10% of nosocomial infections in the USA and 2% in Europe are caused by this pathogen, with these frequencies almost doubling in Asia and the Middle East. Furthermore, around 45% of *A. baumannii* isolates in global terms exhibit MDR (i.e. resistance to at least three classes of antibiotics), with local rates rocketing to 70% in Latin America and the Middle East.^4–7^ Due to this, *A. baumannii* has been included among the most concerning MDR pathogens under the acronym ESKAPE (*Enterococcus faecium*, *Staphylococcus aureus*, *Klebsiella pneumoniae*, *A. baumannii*, *Pseudomonas aeruginosa* and *Enterobacter* spp.).^8^ Moreover, a WHO report highlighted carbapenem-resistant *A. baumannii* as a priority pathogen, for which novel therapeutic approaches urgently need to be developed.^9^

The recalcitrance of this species to treatment is due to its capacity for resistance and persistence,^4^ aided by its multiple MDR mechanisms. These include the cell envelope as a barrier, multidrug efflux systems and mutations in genes coding for porins and antibiotic targets (e.g. ribosomal proteins, PBPs, DNA replication enzymes and the lipid A biosynthetic pathway), as well as enzymes that degrade/inactivate antibiotics.^3^ Oftentimes, these features can spread among the population through mobile genetic elements and the ability of *A. baumannii* to be naturally competent.^3,10–12^

With technological advances, genome-editing tools have evolved, allowing precise genome editing (i.e. insertions and deletions), from a single nucleotide to dozens of kilobases. However, this progress is often uneven, with tools being developed in a biased way for a few well-established model organisms. In the case of *A. baumannii*, many simple targeted genetic tools have been adapted for use in model strains of this pathogen (reviewed by Sykes *et al.*^13^). Mutagenesis in *A. baumannii* was firstly approached by gene disruption by plasmid insertion in a single recombination event and mutation by antibiotic resistance marker insertion.^14^ Next-step strategies include recombineering-based gene disruption followed by removal of the selection marker by site-specific recombination, allowing the use of the same marker for subsequent rounds of mutation to construct multiple mutants.^15^ Even more refined, some protocols allow scarless gene modification by double recombination aided by a counterselectable marker, with strategies taking advantage of the ability of *A. baumannii* to be naturally competent.^16–18^ Moreover, after the emergence of clustered regularly interspaced short palindromic repeats (CRISPR)-Cas systems as a molecular biology tool, a CRISPR-based two-plasmid system for genome editing and a CRISPR interference (CRISPRi) kit for knocking down gene expression have been developed for *A. baumannii*.^19,20^

However, depending on the purpose they are intended for, these genetic editing methods can have some limitations. Gene disruption is not always desirable due to the limited amount of selection markers available and possible polar effects within operons. Strategies including marker removal are usually based on site-specific recombinases that leave a scar in the genome.^15,21^ However, this recombinogenic sequence may cause genomic instability after successive rounds of mutation.^22^ These drawbacks can be prevented by counterselection-mediated scar-free strategies, which allow more complex genome manipulation (i.e. targeted point mutations, domain truncations, allele exchange, deletion of whole clusters), but counterselection (usually based on sucrose sensitivity conferred by *sacB*, which is often unstable in *A. baumannii*) frequently requires passaging under pressing selection and tedious screening for clones that underwent a second recombination event.^16,23^ Furthermore, the current tools are mainly developed for model *A. baumannii* strains, which can be less representative compared with the prevalent clinical isolates. Another major limitation to the application of these tools is that MDR *A. baumannii* strains are resistant to many of the selection markers used in these protocols.^13^

In our efforts to implement state-of-the-art methodologies for standardization of genome editing in non-model MDR *A. baumannii* strains, we have adapted an accelerated highly efficient SceI-based mutagenesis method,^24–27^ developed and optimized for *Pseudomonas putida*,^22,28^ to MDR *A. baumannii* AB5075.^5^ For this, we have modified the two plasmids used in this system with selectable markers that can be used in this strain and subsequently adapted the protocol pipeline. As a proof of concept, we have constructed an in-frame deletion mutant in *craA*, a gene encoding a dedicated chloramphenicol-specific efflux pump. Afterwards, we have attempted to address the function of *cmlA5*, a putative plasmid-borne chloramphenicol efflux pump-coding gene inferred from homology, by comparison with the *craA* mutant. As a result, we have validated the utility of this system for scar-free chromosomal and plasmid editing in *A. baumannii* AB5075.

## Materials and methods

### Bacterial strains and culture media

*A. baumannii* AB5075 (VIR-O colony morphotype),^5,29,30^ its derivate mutants and *Escherichia coli* host strains (DH5ɑ and DH5ɑ λ*pir*) were routinely grown in liquid or solid LB (Miller) at 37°C (180 rpm or static, respectively).^22,31^ When necessary, LB was supplemented with kanamycin (25 mg/L), ampicillin (100 mg/L), apramycin (60 mg/L for *E. coli*, 200 mg/L for *A. baumannii*), tetracycline (5 mg/L) or tellurite (6 mg/L for *E. coli*, 30 mg/L for *A. baumannii*). A summary of strains used in this work is shown in Table S1, available as Supplementary data at *JAC* Online.

### Plasmid construction

A list of plasmids and primer sequences used in this work can be found in Table S1. All plasmid derivatives were constructed using standard restriction-based molecular cloning.

pEMG-Tel (pEMGT) was constructed by cloning a DNA fragment from pMo130-TelR (Addgene, #50799) (bearing the Tel resistance marker) digested with SmaI in pEMG cut with AflIII and blunted with Klenow.^16,22^ For construction of pSW-Apr and pSW-Tc, PCR fragments amplified from pFLAG-attP (Addgene, #110095) with primers Apr fw/Apr rv and from pSEVA524 with primers tetA fw/tetA rv,^32^ respectively, using Q5 High-Fidelity Master Mix (New England Biolabs) were cloned into pSW-I digested with ScaI.^22^

For in-frame deletion of *craA* (ABUW_0337) and *cmlA5* (ABUW_4059), pEMGT-craA and pEMGT-cmlA5 were constructed. For pEMGT-craA, 1 kb upstream and downstream homologous regions were amplified from purified AB5075 genomic DNA with primers craA up fw/craA up rv and craA down fw/craA down rv, respectively, and assembled together by joining PCR. The same procedure was followed for assembly of the *cmlA5* deletion construct using primer pairs cmlA5 up fw/cmlA5 up rv and cmlA5 down fw/cmlA5 down rv. Both constructs were cloned into pEMGT digested with SmaI.

Constructs for Δ*craA* complementation experiments were generated by amplifying the *craA* coding region plus the upstream homologous regions (primer pair craA up fw/craA rv) and the upstream region alone as a control (primer pair craA up fw/craA up rv) and cloning either of them in pEMGT digested with SmaI. The resulting plasmids were designated as pEMGT-up-craA and pEMGT-up, respectively. Complemented strains were constructed by conjugating either pEMGT-up-craA or pEMGT-up as a control and selecting a single recombination event.

All plasmid derivatives were checked by colony PCR using DreamTaq Green PCR Master Mix (Thermo Fisher), restriction patterns and eventually by Sanger sequencing.

### Triparental mating

For transfer of plasmid DNA into *A. baumannii* AB5075 and derivative strains, a standard triparental mating protocol was followed, using pRK2013 (in a DH5ɑ host) as helper plasmid and a DH5ɑ or a DH5ɑ λ*pir* donor bearing the plasmid of interest.^33^ A detailed mating protocol is provided in Text S1. When necessary, DNA deletions were assessed by colony PCR and eventual Sanger sequencing from PCR-amplified genomic DNA. Conjugation frequency was calculated as the number of transconjugant colonies divided by the number of viable cells.

### Antibiotic disc diffusion assay (DDA)

Antibiotic susceptibility assays were performed in cation-adjusted Mueller–Hinton (CAMH) medium (pH 7.4, CaCl_2_ 2 mM, MgCl_2_ 1 mM) (Sigma–Aldrich). Overnight cultures of *A. baumannii* AB5075 or the respective mutant derivatives were diluted to 0.5 McFarland units in CAMHB and spread with a cotton swab on CAMH agar plates. When plates were dry, chloramphenicol, amikacin or tobramycin discs (Oxoid) were placed in the middle of the CAMH agar plate. Plates were incubated at 37°C for 24 h before measuring the diameter of the inhibition zone. Results are shown as averages of three biological replicates.

### MIC determination

Saturated overnight cultures were diluted in PBS to get an OD_600_ of 0.2. Cells were washed three times and resuspended in 1.2 mL of CAMHB. Ten 2-fold serial dilutions of each antibiotic, starting with 2500 μg/mL, were prepared in CAMHB. In order from highest to lowest antibiotic dilution, cell suspensions and antibiotic-supplemented CAMHB were mixed in a 1:1 proportion in a 96-well plate. The plate was then incubated at 37°C, 200 rpm. MICs were assessed by visual examination, defining them as the lowest antibiotic concentration that led to the absence of visible bacterial growth.

### Data analysis

For every experiment, three independent replicates were performed. Results are shown as averages of the three measurements (±SD) or as representative images of the replicates. Result representation and statistical analyses were performed using GraphPad Prism 9.

## Results and discussion

### Rationale of the strategy

To adapt an efficient genome-editing system for MDR *A. baumannii* AB5075, we built our strategy on that developed by Martínez-García and de Lorenzo^22^ for *P. putida*, further optimized to an accelerated version at the Nickel laboratory.^28^ To perform this strategy, plasmids pEMG and pSW-I needed to be used.^22^ pEMG is a cloning suicide vector bearing two target sites for the endonuclease SceI flanking its polylinker. Once the homologous regions flanking the desired modification are cloned into pEMG, the resulting plasmid is transferred to the target strain and integration in the genome is selected. Subsequently, the broad-host-range pSW-I plasmid, with the SceI coding gene under an inducible XylS-dependent promoter, is introduced in the co-integrate strain. Inducing the expression of *sceI* triggers a double-strand break in the genome that is eventually repaired by homologous recombination, generating the reversion to the parental strain genotype or the desired mutation. Apart from improvements to make the screening more efficient, Wirth *et al*.^28^ introduced on-plate induction of *sceI* expression, reducing the second recombination to one single mating and selection step.

**Figure 1.**
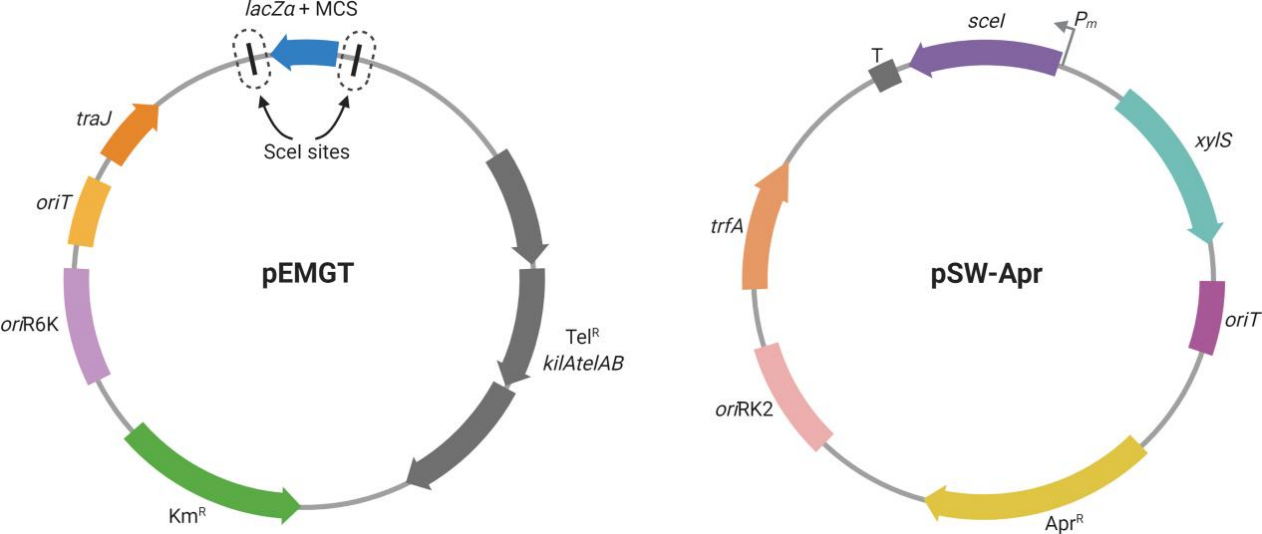
Schematic representation of plasmids pEMGT and pSW-Apr. All relevant features borne in each plasmid are presented and named. SceI target sites in pEMGT are circled in dotted lines. Adapted from ‘Custom Plasmid Maps 2’, by BioRender.com (2022). Retrieved from https://app.biorender.com/biorender-templates. This figure appears in colour in the online version of *JAC* and in black and white in the printed version of *JAC*.

In the case of *A. baumannii* AB5075, one of the disadvantages for its genetic manipulation is its resistance to most available antibiotic selection markers, including those in pEMG and pSW-I. Hence, we constructed a pEMG derivative bearing a tellurite resistance cassette as well as its original kanamycin resistance gene, obtaining pEMG-TelR (pEMGT) as a result (Figure 1). For the second part of the strategy, we produced two variants of the pSW-I plasmid, each bearing either an apramycin resistance marker or a tetracycline resistance gene, namely pSW-Apr (Figure 1) and pSW-Tc, respectively. To validate the method and demonstrate its versatility and robustness, we attempted the construction of scar-free mutants in the chromosome-encoded gene *craA* and the plasmid-borne gene *cmlA5*. *craA* (identified in AB5075 by sequence similarity to the *craA* orthologue characterized in *A. baumannii* ATCC 17978) is an efflux pump previously thought to be specific to chloramphenicol.^34,35^ However, it was recently shown to have a broader substrate range.^36^ On the other hand, *cmlA5* is a putative chloramphenicol efflux pump inferred from homology and encoded within resistance island 2 (RI2) on the native plasmid, p1AB5075.^29^

### Deletion of the chromosomally encoded craA

**Figure 2.**
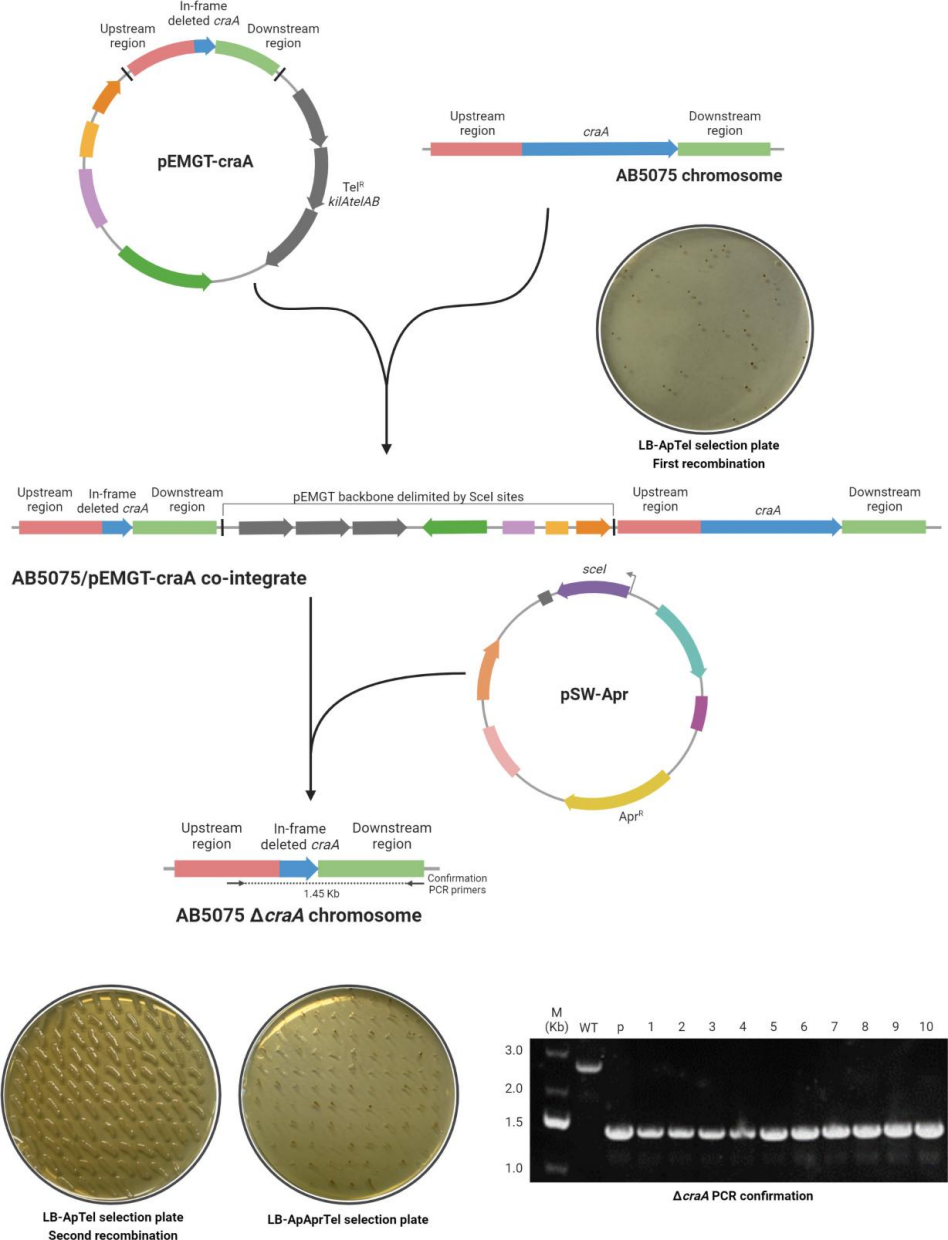
Schematic outline of the genome-editing strategy adapted for *A. baumannii* AB5075 applied to the deletion of *craA*. Plasmid features are represented in Figure 1. When indicated, LB agar plates were supplemented with ampicillin 100 mg/L (Ap), apramycin 200 mg/L (Apr) and/or tellurite 30 mg/L (Tel). For confirmation of *craA* deletion, colony PCR was performed using primers craA fw seq and craA down rv. As controls, WT AB5075 (WT) and pEMGT-craA (p) were used. M, DNA molecular weight marker, with band sizes indicated (kb). For simplicity, only the events occurring if the first recombination happened in the upstream homologous region is shown. Created with BioRender.com. This figure appears in colour in the online version of *JAC* and in black and white in the printed version of *JAC*.

For the first trial of this genome-editing method, we attempted the construction of an in-frame deletion mutant in *craA* (ABUW_0337). A visual outline of the strategy can be followed in Figure 2. Once the pEMGT derivative bearing the flanking homologous regions of *craA* was constructed (pEMGT-craA), it was conjugated into the AB5075 parental strain and transconjugants bearing the plasmid inserted by recombination were selected in the presence of tellurite. Five candidates were confirmed to carry the plasmid integrated into the chromosome by PCR (Figure S1), and transconjugants appeared with a frequency of 10^−8^.

Three colonies were selected from the candidates and brought forward for performing the second recombination event. To check the effectiveness of both pSW-Apr and pSW-Tc in forcing the second recombination event, both of them were transferred by mating in biological triplicates to the AB5075-pEMGT-craA parental strain and transconjugants were selected in the presence of either antibiotic. We attempted the on-plate *sceI* induction by adding the inducer 3-methylbenzoate (3MB) to the selective plates. However, the presence of this compound affected *A. baumannii* growth (Figure S2). Nevertheless, this strategy has been applied before without addition of the inducer,^37–39^ which also proved successful for *A. baumannii* AB5075. In the case of pSW-Apr recipients, clear individual colonies grew with a frequency around 10^−4^. However, although pSW-Tc recipients grew with a similar frequency, colonies appeared with a mucoid phenotype (which we had previously observed when selecting tetracycline resistance) that made selection difficult (Figure S3).

To assess the second recombination, we screened for the loss of tellurite resistance. This screening resulted in 98.0% ± 1.7% of clones that achieved a second recombination triggered by the presence of pSW-Apr (Figure 2) and 72.3% ± 3.2% of clones by pSW-Tc.

To select a double recombinant carrying the in-frame deletion of *craA* instead of a reversion to WT genotype, 10 random candidates among all the pSW-Apr transconjugants were streaked to obtain individual colonies and analysed by PCR. Although the theoretical probability of obtaining a second recombination toward WT configuration or deletion is 50%, the screening resulted in 100% deletion frequency in this case, according to the size of the PCR product, indicating the high efficiency of this mutagenesis strategy.

As a final step in the protocol, the resulting mutant strain had to be cured of pSW-Apr. For this, one mutant clone was inoculated in LB broth in the absence of apramycin and two passages were given after the cultures reached stationary phase. After this, individual colonies were isolated and screened for apramycin-susceptible clones. Chromosomal deletion was validated by sequencing (Figure S4, File S1). Also, since AB5075 bears three native plasmids (p1AB5075, p2AB5075 and p3AB5075), we checked their maintenance after the first recombination event and after the stabilization of a pSW-I derivative by PCR (Figure S5). This showed that all three plasmids can be maintained over the course of this procedure. To facilitate the use of this strategy, a detailed step-by-step laboratory protocol in 7–9 days is shown as Text S1.

### Deletion of plasmid-borne cmlA5, RI2 and p1AB5075 curation

In order to demonstrate the versatility of this mutagenesis toolkit, we attempted the editing of p1AB5075, a native plasmid borne in AB5075. Firstly, we challenged our method by deleting *cmlA5* (ABUW_4059). This gene encodes a putative chloramphenicol efflux pump and is located within RI2, a region encoding multiple aminoglycoside resistance genes.^29,40,41^

For the deletion, we performed a similar strategy as for the mutation of *craA*. Once the respective flanking homologous regions were cloned into pEMGT (pEMGT-cmlA5), the plasmid was transferred to AB5075 and its integration was selected. For the second recombination, we leaned toward using pSW-Apr, given its better performance compared with pSW-Tc. After screening for a second recombination event, we checked 20 candidates by PCR. In this particular case, we found that, whereas 35% of the clones had suffered a second recombination by the homologous regions upstream and downstream *cmlA5* (they gave a PCR of either WT or mutant size), the remaining 65% did not yield any amplification product. This would indicate that either a rearrangement in the plasmid had occurred, removing the region that served as PCR template, or that the whole plasmid had been removed.

**Figure 3.**
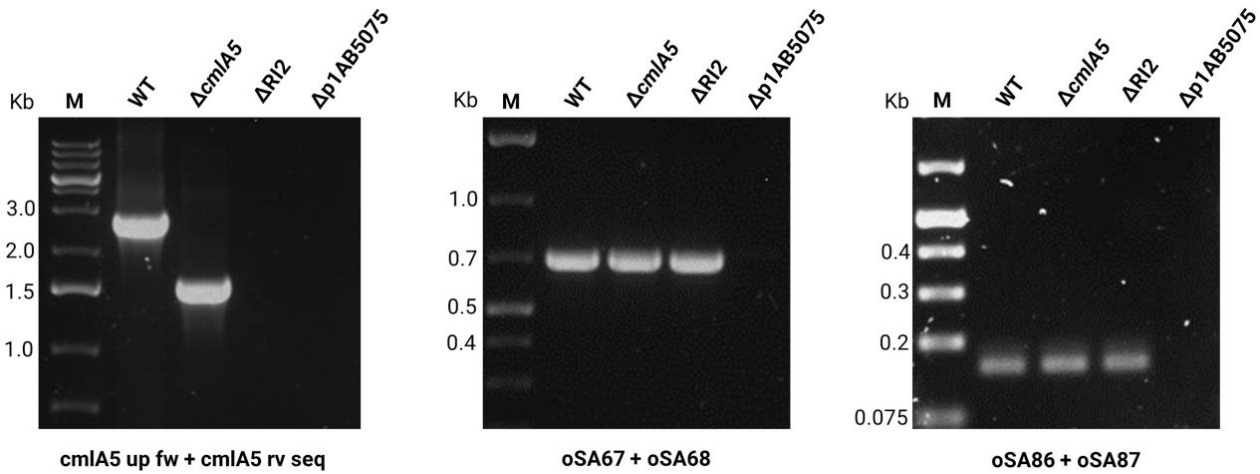
PCR analysis to confirm the Δ*cmlA5* deletion and assess the presence of p1AB5075. Genomic DNA extracted from the respective strain was used as template. For confirming the deletion, primer pair cmlA5 up fw/cmlA5 rv seq was used, giving bands of 2.79 kb for the WT and 1.55 kb for the Δ*cmlA5* deletion mutant. The presence or absence of p1AB5075 was assessed with primer pairs oSA67/oSA68 and oSA86/oSA87, which would give PCR products of 0.7 and 0.16 kb, respectively.^44^ In the case of the p1AB5075-cured strain, no amplification was observed for any of the primer pairs. Scission of RI2 from p1AB5075 (ΔRI2), supported by Figure S6, explains the absence of PCR product using the primers to detect *cmlA5* and compared with the amplification with primers to confirm the presence of p1AB5075. M, DNA molecular weight marker, with band sizes (kb).

RI2 comprises a 7.8 kb region in p1AB5075 delimited by two homologous integrase coding sequences.^29,42^ Homologous recombination between the two integrase regions has been documented before, amplifying the copy number of RI2 and producing aminoglycoside heteroresistance.^42^ In our attempts to delete *cmlA5*, the strategy required the introduction of the upstream and downstream homologous region of this gene, by which a recombination can happen to repair the SceI double-strand break. As the SceI target sequences would be inserted between those two pairs of homologous regions, the recombination repair could happen either between the *cmlA5*-flanking regions, deleting the gene, or by the RI2-delimiting regions, thus deleting the whole resistance island. We could validate the latter case by PCR in those clones that did not yield any amplification with the primer pair used to validate the *cmlA5* deletion, but still showed the presence of the rest of p1AB5075 (Figure S6), thus obtaining a ΔRI2 mutant.^29,43^ For those candidates that still did not give a PCR product with the primer pairs used so far, we validated the presence of p1AB5075 by using two primer pairs outside of RI2 previously used to monitor the presence of this plasmid.^44^ This resulted in 20% of the clones not yielding a PCR product with any primer pair, indicating the loss of p1AB5075. One example of different template–primer pair combination indicating presence of *cmlA5*, RI2 and/or p1AB5075 is shown in Figure 3.

Curing native plasmids, usually of unknown function, often involves tedious counterselection screenings.^45–47^ Otherwise, spontaneous plasmid-cured strains can be found serendipitously.^44,48^ Given the high frequency of *A. baumannii* strains bearing multiple native plasmids and the difficulties entailed by mutating and manipulating them, this methodology shows a remarkable potential to facilitate their study.

### Phenotypic characterization of the *Δ*craA and *Δ*cmlA5 mutants

To assess the efficiency of this mutagenesis method, we chose to delete genes linked to antibiotic resistance, whose phenotype can be measured easily. Whereas there are reports about the role of CraA in chloramphenicol resistance,^35,36^ the RI2-encoded CmlA5 has only been annotated as a chloramphenicol efflux pump based on homology (Figure S7).^29,40,41^ The phenotypic characterization and comparison of both mutants would help us elucidate the relative contribution of CraA and CmlA5 to antibiotic resistance in AB5075.

**Figure 4.**
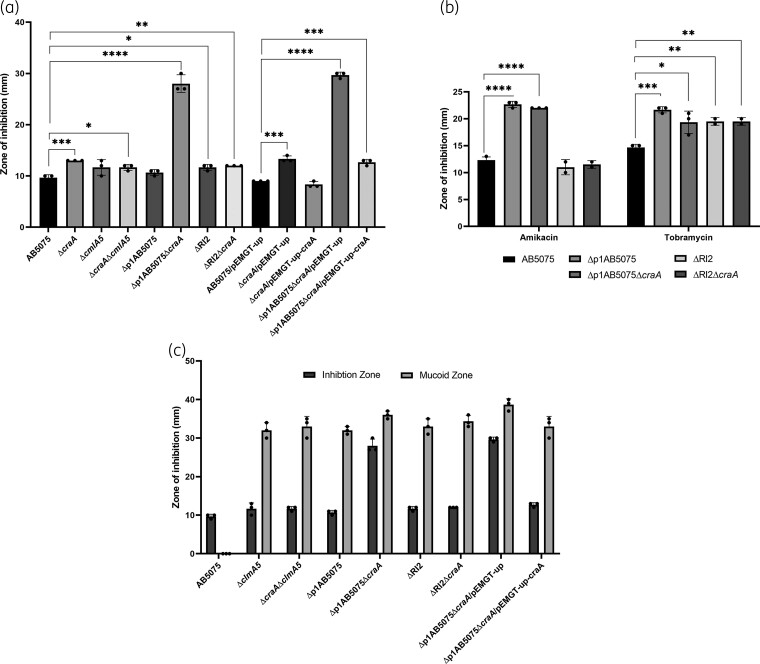
Quantification of antibiotic resistance and mucoid phenotype of the multiple mutant strains compared with AB5075. Quantifications were performed by DDAs using chloramphenicol (50 µg), amikacin (30 µg) and tobramycin (10 µg) discs according to the experiment. (a) Chloramphenicol resistance was measured for all mutant strains generated and compared with that of the WT AB5075. The susceptibility phenotypes observed for the Δ*craA* and Δp1AB5075Δ*craA* mutants were complemented by reintroduction of the *craA* coding sequence as compared with the WT and the parental strains bearing the control construction (see Materials and methods). (b) In the case of the mutants affecting RI2 or the whole p1AB5075 plasmid, as well as their combinations with the Δ*craA* mutation, resistance was also assessed for the aminoglycosides amikacin and tobramycin. (c) For the mutants that produced it, the mucoid zone observed around chloramphenicol discs was measured and compared with the zone of inhibition using the WT strain (no mucoid zone formed) as control. The average zone of inhibition in millimetres (mm) measured from three biological replicates ( ±SD) is shown. Statistical significance was assessed from *P* values obtained from a *t*-test (* = *P* ≤ 0.05, ** = *P* ≤ 0.01, **** = *P* ≤ 0.0001).

For their characterization, we assessed chloramphenicol resistance by DDAs and MIC measurements comparing both deletion mutants to the WT [Figure 4(a), Table 1, Figure S8]. The DDAs showed that the Δ*craA* mutant was the only one with a significantly increased susceptibility to chloramphenicol (MIC of 50 mg/L compared with 200 mg/L for AB5075). This increased susceptibility could be reverted by complementing the Δ*craA* mutation [Figure 4(a), Table S2]. The MIC assays revealed an increase in susceptibility for the Δ*cmlA5* mutant (100 mg/L). In the case of a Δ*craA*Δ*cmlA5* double mutant, it intriguingly showed a similar susceptibility to the Δ*cmlA5* mutant strain rather than an additional susceptibility. This suggests there is not an additive effect between these two chloramphenicol resistance genes, but they would be related in an indirect manner. Altogether, this confirms the role of CraA in chloramphenicol resistance in AB5075, and suggests a milder contribution of CmlA5.

**Table 1.** MIC for the Δ*craA* and Δ*cmlA5* mutants and the p1AB5075-cured strain (Δp1AB5075) compared with the WT AB5075.

	MIC (mg/L)		
	Amikacin	Chloramphenicol	Tobramycin
AB5075	100	200	50
Δ*craA*	N/A	50	N/A
Δ*cmlA5*	N/A	100	N/A
Δ*cmlA5*Δ*craA*	N/A	100	N/A
Δp1AB5075	3.125	50	<0.78125
Δp1AB5075Δ*craA*	1.5625	3.125	1.5625
ΔRI2	100	100	6.25
ΔRI2Δ*craA*	100	100	6.25

MICs were assessed in CAMHB. Antibiotic 2-fold dilutions ranging from 200 to 0.391 mg/L in the case of chloramphenicol and 200 to 0.781 mg/L in the case of amikacin and tobramycin were used. The MIC was assessed as the first concentration that showed no visual growth (Figure S8). Three biological replicates were conducted. N/A: not applicable.

### Phenotypic characterization of the ΔRI2 mutant and the p1AB5075-cured strain

Apart from *cmlA5*, RI2 encodes four aminoglycoside resistance genes. Due to this, we aimed to quantify the resistance of the plasmid-related mutants (ΔRI2, Δp1AB5075) to the aminoglycosides amikacin and tobramycin [Figure 4(b), Table 1, Figure S8].^29,44^ We observed that the ΔRI2 mutant demonstrated an increased susceptibility to both aminoglycosides. Furthermore, curing the WT strain of the p1AB5075 led to a much greater increase in susceptibility to both amikacin and tobramycin compared with the sole deletion of RI2. This can be explained by the presence of multiple aminoglycoside resistance genes at other locations outside of RI2 in p1AB5075.^29^ The deletion of *craA* in the plasmid-related mutant did not affect aminoglycoside resistance [Figure 4(b), Table 1].

Regarding chloramphenicol resistance of the p1AB5075-related mutants, we could observe different levels of resistance according to the MIC results (Table 1), all of them showing greater susceptibility than the WT. Apart from the inhibition zone, a zone of mucoid, translucent biomass appeared in chloramphenicol DDA assays, which was also observable for the Δ*cmlA5* single mutant [Figure 4(c), Figure S9]. Strikingly, the Δp1AB5075Δ*craA* double mutant exhibited a zone of inhibition of approximately double the diameter of that observed for the Δ*craA* single mutant or the p1AB5075-cured strain. Moreover, it covered an equivalent area to the mucoid zone shown by the rest of the mutant strains. This phenotype could be complemented by reintroducing the *craA* coding region [Figure 4(a), Figure S10, Table S2], recovering the inhibition zone and mucoid phenotype of the Δp1AB5075 single mutant. When biomass from the mucoid zone was restreaked in the absence of chloramphenicol, the phenotype reverted to non-mucoid, opaque colony morphotype in all cases, indicating that the phenotype was not caused by additional mutations nor phase variation.^30^ This suggests there might be an interplay between CraA and p1AB5075 in conferring full resistance to chloramphenicol. Unravelling this plasmid–chromosome regulatory interplay may shed light on the capacity of this pathogen to overcome chloramphenicol treatment in the clinic and will be the focus of future work.

Apart from its role in chloramphenicol resistance,^35^*A. baumannii* CraA was recently shown to have a broader substrate range, including other chloramphenicol derivates and biocides, such as chlorhexidine, benzalkonium and dequalinium.^36^ Consequently, it was postulated to be closer in function to the multidrug efflux pump MdfA, although differing in the substrate recognition mechanism.^36^ However, this broadening in the substrate specificity does not reach aminoglycosides according to our results, as we could not see an increase in susceptibility in the Δ*craA* mutant, but only in the absence of RI2 or p1AB5075. Furthermore, this higher susceptibility would not fully manifest in the absence of RI2, which is in agreement with the presence of other aminoglycoside resistance genes outside this region and within p1AB5075.^29^ Regarding chloramphenicol resistance, we show that mutations affecting p1AB5075, even the sole mutation of *cmlA5*, lead to the formation of a mucoid zone. Previously, it was reported that chloramphenicol may trigger a mucoid phenotype by inducing capsule production.^49^ Furthermore, the greater inhibition zone of the Δp1AB5075Δ*craA* compared with the Δp1AB5075 and the Δ*craA* single mutants suggests a synergistic effect between p1AB5075 and CraA in chloramphenicol resistance. However, the implication of p1AB5075 in this phenotype remains to be understood.

All in all, we showcase an efficient and robust genome-editing toolkit that can be used to modify both the chromosome and the native plasmids harboured by MDR *A. baumannii*.

## Supplementary Material

dkac328_Supplementary_Data

## Data Availability

All plasmids are available through request to the corresponding author.

## Supplementary data

Figures S1 to S10, Tables S1 and S2, Text S1 and File S1 are available as Supplementary data at *JAC* Online.
